# Development of Prediction Model to Predict the Compressive Strength of Eco-Friendly Concrete Using Multivariate Polynomial Regression Combined with Stepwise Method

**DOI:** 10.3390/ma15010317

**Published:** 2022-01-02

**Authors:** Hamza Imran, Nadia Moneem Al-Abdaly, Mohammed Hammodi Shamsa, Amjed Shatnawi, Majed Ibrahim, Krzysztof Adam Ostrowski

**Affiliations:** 1 Department of Construction and Project, Al-Karkh University of Science, Baghdad 10081, Iraq; 2 Department of Civil Engineering, Najaf Technical Institute, Al-Furat Al-Awsat Technical University, Najaf Munazira Str., Najaf 54003, Iraq; nadia.material@gmail.com; 3 Department of Architecture, Faculty of Engineering, University of Kufa, Najaf 54002, Iraq; mohammedh.shamsah@uokufa.edu.iq; 4 Applied Earth Sciences and Environment Department, Institute of Earth and Environmental Sciences, Al Al-Bayt University, Mafraq 25113, Jordan; Shatnawi@aabu.edu.jo; 5 Department of Geographic Information Systems and Remote Sensing, Institute of Earth and Environmental Sciences, Al Al-Bayt University, Mafraq 25113, Jordan; majed.ibrahim@aabu.edu.jo; 6 Faculty of Civil Engineering, Cracow University of Technology, 24 Warszawska Str., 31-155 Cracow, Poland

**Keywords:** machine learning, compressive strength of concrete, ground granulated blast-furnace slag, recycled concrete aggregate, multivariate polynomial regression (MPR)

## Abstract

Concrete is the most widely used building material, but it is also a recognized pollutant, causing significant issues for sustainability in terms of resource depletion, energy use, and greenhouse gas emissions. As a result, efforts should be concentrated on reducing concrete’s environmental consequences in order to increase its long-term viability. In order to design environmentally friendly concrete mixtures, this research intended to create a prediction model for the compressive strength of those mixtures. The concrete mixtures that were used in this study to build our proposed prediction model are concrete mixtures that contain both recycled aggregate concrete (RAC) and ground granulated blast-furnace slag (GGBFS). A white-box machine learning model known as multivariate polynomial regression (MPR) was developed to predict the compressive strength of eco-friendly concrete. The model was compared with the other two machine learning models, where one is also a white-box machine learning model, namely linear regression (LR), and the other is the black-box machine learning model, which is a support vector machine (SVM). The newly suggested model shows robust estimation capabilities and outperforms the other two models in terms of *R*^2^ (coefficient of determination) and RMSE (root mean absolute error) measurements.

## 1. Introduction

Most emerging nations have experienced a tremendous expansion in industrialization and urbanization in recent decades, resulting in a large increase in demand for natural raw resources. The building sector has a greater negative influence on the environment since it produces a large amount of waste coming from construction and demolition (C&D) with no viable alternatives; it also uses a considerable quantity of energy and natural raw materials. Furthermore, the by-products, such as blast-furnace slag, silica fume, fly ash, and ferrochrome slag, generated by industrial activities and others contribute to difficulties such as trash-disposal land scarcity and rising waste treatment costs before the dumping. As a result, economic activities should be pursued to keep the earth’s ecology in balance [1]. Because concrete is widely used in the construction sector, reducing the negative effects of concrete on the environment can help to ensure the industry’s long-term viability. This may be accomplished by using the above-mentioned industrial by-products and C&D waste as a replacement material for concrete elements such as cement and aggregates. C&D wastes have been recommended for use as a substitute for natural coarse aggregates (NCA) in concrete, which are called recycled coarse aggregates (RCA). According to research [1,2], recycled aggregate concrete (RAC) has poorer durability and mechanical performance than natural aggregate concrete (NAC). This is because RCA’s quality is inferior to NCA’s. Recycled concrete aggregate is typically prepared using industrial jaw crushers. This most frequently used method of aggregate preparation is associated with the formation of microcracks in aggregate grains, which consist of natural aggregate and a cement matrix (which may contain various additives apart from cement). A weaker interfacial transition zone is generated because of the presence of connected mortar on the RCA and renders it porous. Various improvement approaches for increasing the properties of RAC have been proposed in the literature. One of the best cost-effective approaches to enhance the qualities of RCA and obtain RAC that is similar to NAC is by adding supplementary cementitious materials [3,4]. Additional cementitious ingredients including metakaolin (MK), ground granulated blast-furnace slag (GGBS), silica fume (SF), and fly ash (FA) have been used in blended cement or Portland cement concretes for many years [5,6,7]. By providing a denser matrix, the use of GGBFS to replace ordinary Portland cement partially increases concrete strength and durability, extending the service life of concrete structures. Although it is commonly acknowledged that combining FA and GGBS with normal fineness lowers concrete’s early strength, they offer long-term benefits such as reduced alkali–silica reaction expansion, reduced porosity and permeability, and improved workability [8,9].

The concrete compressive strength test displays the concrete’s characteristic strength, and is typically performed to examine the concrete’s working stress after 3 days, 7 days, 28 days, or 90 days [10]. The compressive strength test is a standard test that is used during structural design to assess the maximum working stress of concrete. It is a measure of quality control of concrete production in a factory or workshop.

Since eco-friendly concrete has more forecasting parameters, such as GGBFS or RCA, it is relatively hard for traditional linear regression to predict its flexural and compressive strength. Machine learning approaches can be used to tackle the challenge of forecasting the strength of eco-friendly concrete. Multivariable statistical approaches are a useful tool for better understanding and interpreting difficult data [11]. Multivariate polynomial regression (MPR) has been used for years to handle several civil engineering difficulties, notably in building material fields [12,13,14]. Because it is a representational model, such MPR models have an advantage over black-box models such as ANNs in that they can be more easily studied using techniques such as graphical approaches and sensitivity analysis and by applying variable importance ratios. Because of these capabilities, the MPR can be a highly useful instrument.

In the last few years, many researchers have developed different prediction models to estimate the compressive strength of eco-friendly concrete [15,16,17,18,19,20,21,22]. To build a prediction model for the CS that contains RAC, two types of hybridized machine learning methods (an interval type-2 fuzzy inference system (IT2FIS) and type-1 fuzzy inference system (T1FIS)) were used [15]. According to the findings, the IT2FIS model surpassed the T1FIS model in performance. Moreover, based on a meta-heuristic search of sociopolitical algorithms (i.e., ICA), the 28-day RAC concrete compressive strength was investigated using four artificial intelligence techniques [16]. The results reveal that the suggested ICA-XGBoost model outperformed the other models. In this study [17], RAC compressive strength and its optimal mixture design were predicted by machine learning models. The results demonstrated that the generated models, including deep learning, Gaussian processes, and gradient boosting regression, obtained reliable predictive performance, with the gradient-boosting regression trees outperforming the others. A prediction model to estimate the compressive strength of RAC was built using a convolutional neural network in another study [18]. The construction of the deep learning model was accompanied by experimental work. The convolutional neural network model was compared with two other models—a back propagation neural network and a support vector machine—indicating that the convolutional neural network can predict the compressive strength of RAC better than the others. The paper used [19] three different machine learning models to predict the compressive strength of eco-friendly concrete that contains GGBFS; these were artificial neural network (ANN), support vector machine (SVM), and multiple linear regression (MLR). Using k-fold cross-validation, the ANN and SVM approaches were compared with MLR, revealing that the artificial intelligence methods performed better. The study [20] used a random forest algorithm to predict ground granulated blast-furnace slag concrete (GGBFSC) without adding RCA to the concrete mixtures. The prediction model was built using several associated variables for the concrete material, such as curing temperature (T), superplasticizer, fine aggregate (FA), coarse aggregate (CA), water-to-binder ratio (w/b), water content (W), GGBFSC-to-total-binder ratio (GGBFSC/B), and so on (SP). Using restricted input parameters, the RF model gave excellent results in the prediction of CS, depending on the prediction precision achieved. Adding GGBFS to concrete mixtures, paper [21] also suggested a strategy based on random forest (RF) for forecasting concrete compressive. Based on the result obtained, the RF algorithm showed a high prediction accuracy and may be used to reduce experimental costs. In this study [22], fuzzy logic and artificial neural network models for prediction of long-term effects of ground granulated blast-furnace slag on compressive strength of concrete under wet curing conditions have been developed. Artificial fuzzy logic systems and neural networks have demonstrated high promise for predicting long-term effects of ground granulated blast-furnace slag on concrete compressive strength in training and testing models.

## 2. Research Significance

Most of the above-mentioned studies performed machine learning and artificial intelligence models to forecast the compressive strength of eco-friendly concrete. Despite their excellent performance in the process of predicting the mechanical properties of concrete, they work as a complex system that is hard to implement and interpret. The MPR model suggested in this study is significantly more visible and straightforward for academics to utilize, unlike existing black-box algorithms such as artificial neural networks. Moreover, previous researchers built a prediction model for compressive strength for eco-friendly concrete that contains either RCA as a replacement material for natural aggregate or GGBFS as a replacement material for ordinary cement, but not both. Unlike in other research, in this study a prediction model for the compressive strength of concrete that contains both RCA and GGBFS materials is proposed.

## 3. Materials and Methods

### 3.1. System Methodology

**Figure 1:**
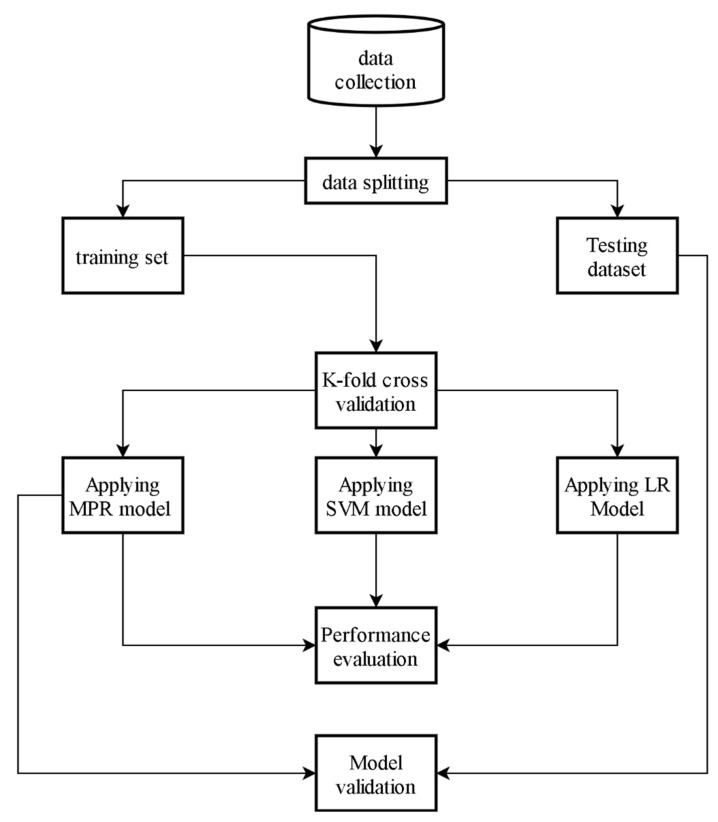
Proposed system methodology for eco-friendly concrete compressive strength prediction.

An efficient technique applicable in this work that allows us to forecast the compressive strength of eco-friendly concrete was constructed. The system technique is depicted in Figure 1. In this framework, we compared the performance of the MPR method to estimate the compressive strength of eco-friendly concrete with the LR (white-box algorithm) and SVM (black-box algorithm) models. The initial stage of the system, as indicated in Figure 1, is to collect experimental data from prior studies. The technique of dividing data into two portions, which are training and testing datasets, is the second stage. The third stage is splitting our training dataset into fivefold cross-validation in order to evaluate the performance of the three models. The fourth stage is applying all three models on k-fold cross-validation. The fifth stage is evaluating the performance of the MPR model with the two other machine learning models using well-known performance metrics. Finally, we developed a regression equation from a training dataset and used it on unseen data to validate our model.

### 3.2. Dataset

**Figure 2:**
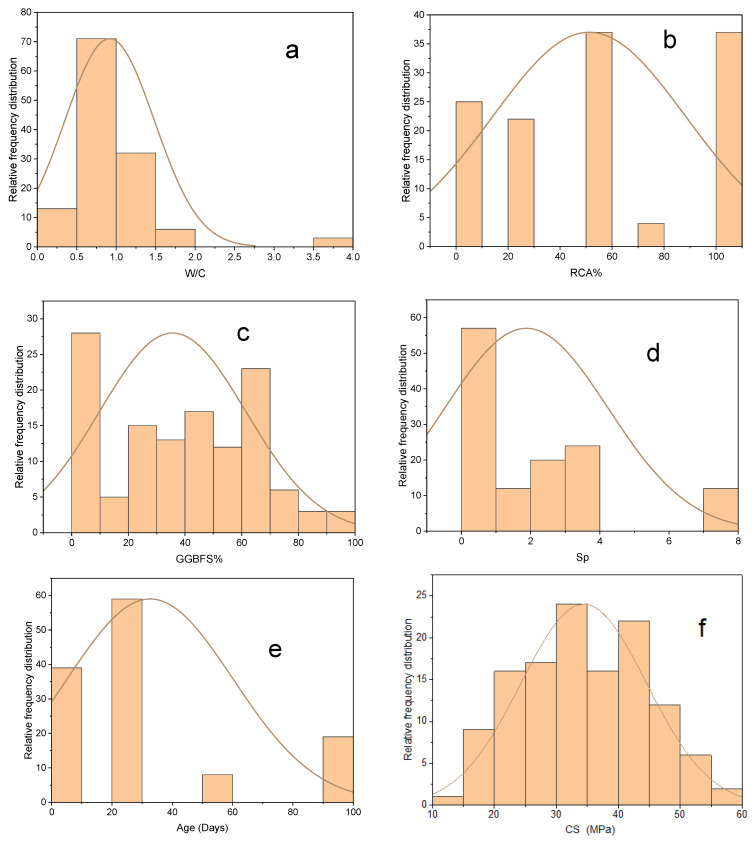
Relative frequency distribution of variables: W/C (**a**), RCA% (**b**), GGBFS% (**c**), Sp (kg) (**d**), Age (**e**), CS (**f**).

The model’s effectiveness is entirely determined by the components and the quantity of data samples utilized. The variables utilized to create models for predicting concrete strength were gathered from the available literature [23,24,25,26,27,28], yielding 125 mix proportions in total. The predictors used from the literature to build the models were: water-to-cement ratio (W/C), recycled aggregate percentage as a replacement material for normal aggregate in the mixture (RAC%), GGBFS percentage of replacement as a replacement material for OPC in the binder (GGBFS%), superplasticizer, and age (days). The target variable was compressive strength (CS) of eco-friendly concrete. Figure 2 depicts the overall distribution of all variables in terms of relative frequency. Table 1 shows the statistical description for the variables.

### 3.3. Data-Splitting Procedure

Following the acquisition of the dataset, we divided it into two parts at random: the testing set and the training set. The training-to-testing-sets ratio in this study was 8:2. We used 120 observations in the training set to train our model, and 25 observations in the testing set to test its performance. A stratified sampling approach was used to choose the training sample from the initial datasets, ensuring that the entire data has a comparable outcome distribution.

### 3.4. MPR Model Development

The relationship between dependent and independent variables was fitted by polynomial regression. The following math formula represents the general equation for the sth-order (*s* > 1) polynomial regression [29]:

(1) $$\hat{y}=w_{0}+w_{1}x+w_{2}x^{2}+w_{3}x^{3}+\ldots +w_{s}x^{s}$$

where *w*_1_, *w*_2_, … *w_s_* are the polynomial regression coefficients, *x* is the input variable, $\hat{y}$ is the output variable, and *w*_0_ is the intercept. When multiple variables are introduced to polynomial regression, it can be named MPR. The following formula represents MPR for a system with *n* input variables and the sth-order (*s* > 1) [29]:

(2) $$\begin{array}{l} \hat{y}=w_{0}+\sum_{l_{1}=1}^{n}w_{l_{1}}x_{l_{1}}+\sum_{l_{1}=1}^{n}\sum_{l_{2}=l_{1}}^{n}w_{l_{1}l_{2}}x_{l_{1}}x_{l_{2}}+\ldots +\\ \sum_{l_{1}=1}^{n}\sum_{l_{2}=l_{1}}^{n}\ldots \sum_{l_{k}=l_{k-1}}^{n}w_{l_{1}l_{2}}\ldots_{l_{k}}x_{l_{1}}x_{l_{2}}\ldots x_{l_{k}} \end{array}$$

The multivariate function (Equation (2)) is a linear function with respect to its coefficients even if the MPR adapts a non-linear model to the data. As a result, when the least-squares approach is utilized, the MPR model has the same solution as the MLR problem. Reducing the sum of squared errors of the expected vs. actual outcome leads to finding the polynomial regression coefficients by least-squares methods.

Integers or fractions might be used as exponents for MPR predictors. Only integer exponents between −3 and +3 were evaluated in this study. Outside of this range, preliminary testing of fractions and integers yielded no significantly improved models. In the form of a general equation (Equation (2)), we used a TaylorFit [30] software to provide a standard mathematical explanation of MPR. MPR incorporates interaction and other nonlinearities, and it is essentially considered an extension of a multilinear regression (MLR). Further, as the number of multiplicands and the number of exponents grows, the number of terms increases. For each candidate term utilized in this investigation, the maximum number of multiplicands was 3. A stepwise algorithm, as follows, was used to build the model:

1. An intercept (average of the dependent variable values) was always the first step in the model. Depending on the permissible exponents and multiplicands selected by the user, the software created terms that best interacted with existing model terms. The terms were ranked by the fit data’s best t-statistics.
2. For a term to be included in the model, two criteria must be met. First, the candidate term should be statistically significant variables of fit. Second, the overall RMSE value of different cross-correlation dataset should be improved. This method lowered the likelihood of overfitting and enhanced the model’s generalizability.
3. After any item was added to the model, the statistical significance of the previously included terms was assessed, and if they were not, they were eliminated.
4. For more possible terms, the above procedure was done iteratively.
5. The model was created using an iterative procedure of introducing and eliminating potential terms from a list of statistically significant terms based on the fit dataset, which also enhanced the RMSE of the test dataset, until the model could no longer be enhanced by introducing or eliminating any individual term.

### 3.5. Cross-Validation

In a limited data sample, one of the best practices is to use a resampling technique called cross-validation to evaluate machine learning models. The process includes only one parameter, k, which specifies the number of groups into which a given data sample should be split. As a result, the process is frequently referred to as k-fold cross-validation. When a precise value for k is specified, it may be substituted for k in the model’s reference, for example, k = 5 for 5-fold cross-validation. In machine learning, we use cross-validation primarily to evaluate the machine learning model’s skill on new data [31]. That would be to employ a small sample to assess how the model will behave in practice when deployed to generate forecasts on data that was not utilized during the model’s training. Compared to other methods, such as a simple test/train division, k-fold is easy to grasp and produces a less optimistic or biased estimate of the model skill.

The following is the general procedure:

1. Randomly shuffle the database.
2. Construct k-groups out of the data.
3. For each distinct group:
  - As a holdout or test data set, use the group.
  - As a training data set, use the remaining groups.
  - Apply a model over the training set and assess it against the test set.
  - Keep the assessment score but discard the model.
4. Using a sample of the model assessment results, summarize the model’s ability.

Notably, every observation in the data sample is allocated to a unique group and remains there throughout the method. In this process, every distinct sample is used to train the model k − 1 times and has one chance to be utilized in the holdout set [32].

The mean of the model skill scores is frequently used to describe the outcomes of a k-fold cross-validation run. The measure of the variance, such as the standard error and standard deviation, is also a good practice for the measure of the model performance.

The choice of k in k-fold cross-validation comes with a bias–variance trade-off. Considering those factors, k-fold cross-validation with k = 10 or k = 5 is typically used, since these numbers have been demonstrated practically to generate test error rate estimates with neither extremely high bias nor remarkably high variation [32]. For evaluation purposes, two machine learning algorithms were also developed in this process. These two algorithms were SVM (black-box model) and LR (white-box model). These models were built using the WEKA (Waikato Environment for Knowledge Analysis) software. The same fivefold cross-validation that was applied to MPR was also applied to the SVM and LR models.

### 3.6. Performance Metrics

Ultimately, we computed the RMSE (root mean square error) and *R*^2^ factors to determine the reliability of each model (with varying degrees of complexity). The RMSE factor (Equation (3)) was used to calculate the average Euclidian distance between actual values and predicted as [33].

(3) $$RMSE=\sqrt{\frac{1}{n}\sum_{i=1}^{n}{\left(y_{i}^{obs}-y_{i}^{pre}\right)}^{2}}$$

where *y_i_^obs^* and *y_i_^pre^* are the actual output and predicted values, respectively. Because the RMSE has similar values as the output variables, it was applied to measure the accuracy of the compressive strength values forecasted by each model (lower RMSE values imply more accuracy). We calculated the *R*^2^ value (Equation (4)), which is the proportion of the dependent variable variance, in addition to the RMSE.

(4) $$R^{2}=\frac{\sum_{i=1}^{n}{\left(y_{i}{}^{obs}-y^{-obs}\right)}^{2}-\sum_{i=1}^{n}{\left(y_{i}{}^{obs}-y_{i}{}^{pre}\right)}^{2}}{\sum_{i=1}^{n}{\left(y_{i}{}^{obs}-y^{-obs}\right)}^{2}}\in [0,1].$$

where *y^−obs^* is the average of all observed data. This metric was to be utilized to determine the nearness of the data to the fitted line. *R*^2^ = 1 shows that the forecast is perfect, whereas lesser values imply that the prediction is less accurate.

Based on the results of fivefold cross-validation cycles, boxplots of RMSE and *R*^2^ were constructed for each MPR, SVR, and linear regression model. Boxplots allow us to display data quartiles (or percentiles), averages, and the median. They also show the skewness and distribution of model findings. Pair plots were created in addition to these boxplots to graphically examine the model’s performance on the validation sample data (testing dataset). Pair plots were created by visualizing the actual values on the X-axis and the estimated value on the Y-axis. A 45-degree line was drawn from the origin to show how the predicted values differed from the real ones in the test dataset.

## 4. Model Result

### 4.1. K-Fold Cross-Validation

**Figure 3:**
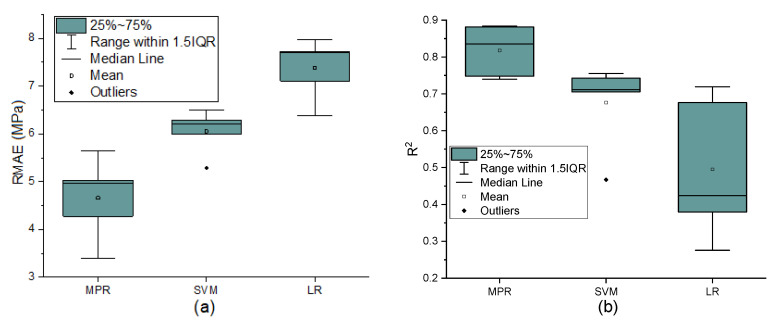
Comparing the performance of MPR, SVM, and LR for five-fold cross-validation training dataset with boxplots: RMSE (**a**), *R*^2^ (**b**).

The boxplots (Figure 3) depict the ranges and variation of the performance measures, *R*^2^, and RMSE, for each ML model’s training performance. These boxplots were created using 100 real values from the training datasets’ 5-fold cross-validation. The minimum, maximum, median, and average for RMSE and *R*^2^ for three machine learning models using the fivefold cross-validation are in Table 2.

Our MPR model clearly surpasses LR and SVM in terms of the median and mean values of the two metrics. The superior performance of nonlinear models was due to the nonlinear interactions of compressive strength with the input variables.

**Figure 4:**
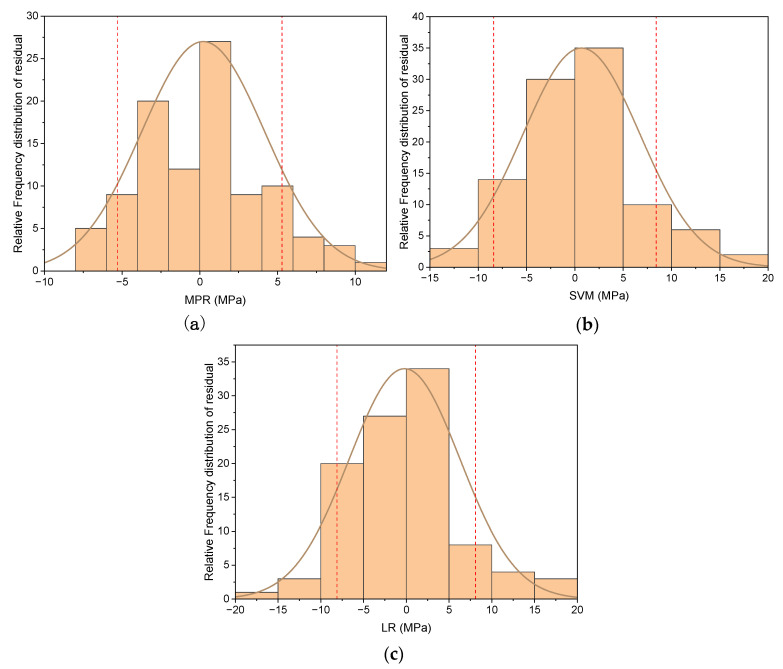
Normal distribution of the residual errors of five-fold cross-validation training dataset. (**a**) MPR model (**b**) SVM model (**c**) LR model.

Additional examination on model stability and dependability is required, in addition to an assessment of the model’s prediction accuracy. The random error for a decent model should follow a normal distribution, with a symmetric behavior following N (σ,0) and with the majority of errors towards the center. The residual distributions and fitted Gaussian functions of the three models are shown in Figure 4. Three models’ residuals follow a normal distribution in general. The dashed red lines represent the 10th and 90th percentiles of the normal distribution of residual, respectively. The 80-percent residuals of the MPR models are between [−5.3, 5.3] based on the distribution patterns, showing that MPR models have greater prediction accuracy than the SVM and LR method. We can notice also that the MPR’s continuity is far superior to that of the SVM and LR models.

### 4.2. Model Validation

After we evaluated the performance of our MPR model with the other two models using five-fold cross-validation, we constructed a prediction formula to estimate the compressive strength of eco-friendly concrete using the training dataset. The final prediction formula is as follows:

CS = (0.001110429806568298 × (W/C)^2^ × (GGBFS%)^3^ × (Age)^−3^) + (−4267.442949761137 × W/C × (Age)^−3^) + (34.78562674882411) + (−0.04850685145004892 × (W/C)^−1^ × RCA%) + (7.39980447082911 × 10^−8^ × (W/C)^−1^ × (RCA%)^−3^ × GGBFS%) + (0.000005453293244435137 × (W/C)^−2^ × (Age)^3^) + (0.21181781054751617 × (W/C)^−3^ × (Sp)^2^); (5)

**Figure 5:**
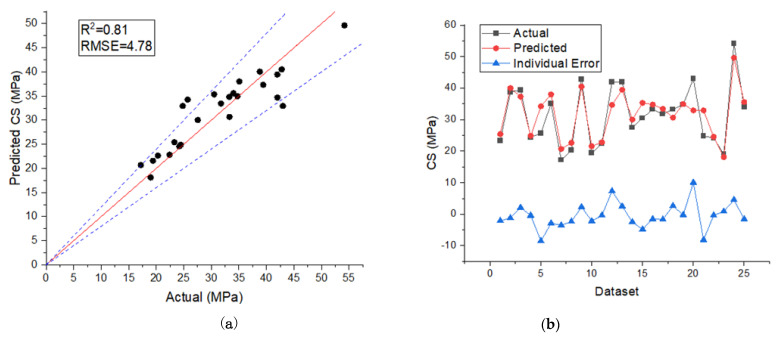
Actual versus predicted values of concrete compressive strength using testing dataset (**a**); model errors between targets and predictions from MPR model technique (**b**).

The final prediction model for the compressive strength obtained from the MPR model was used to estimate the compressive strength of unseen data (testing dataset). Using pair plots, Figure 5a depicts all predicted values of concrete compressive strength versus actual values for the testing data. As we can notice in Figure 4a, the MPR model’s predicted data points are near the 45th degree. This shows that the MPR model can generalize well and predict accurately for samples outside of the train set. For the test data, the RMSE and *R*^2^ between the observed and the projected MPR model values were 4.78 and 0.81, respectively. Figure 5b also shows the distribution of the predicted values of concrete compressive strength, the actual values, and the errors in the MPR model. The average, highest, and lowest error values for the distribution were −0.48, 10.04, and 0.21 MPa, respectively. It is worth mentioning that the mean value of the actual as well as the mean value of the predicted compressive strength of the testing dataset were 31.32 MPa and 31.80 MPa, respectively, while the standard deviation values of both were 9.41 MPa and 7.58 MPa, respectively. The detailed results for each of the dataset testing mixtures and corresponding actual versus predicted values can be seen in Table 3.

### 4.3. Parametric Study

**Figure 6:**
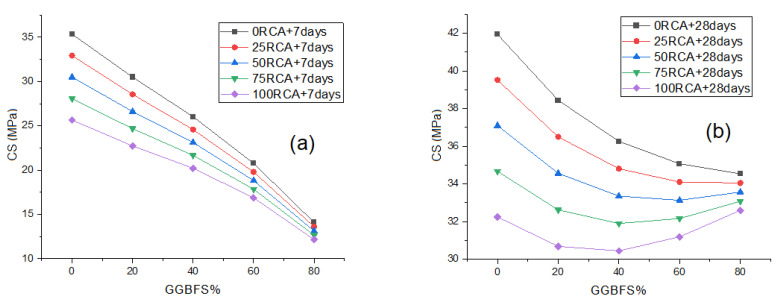
The effect of GGBFS and RAC on compressive strength at early stage (**a**), effect on GGBFS and RAC on compressive strength at late stage (**b**).

A parametric analysis was also performed in this study to determine the effectiveness of the constructed MPR model in predicting the compressive strength trend as the input variables changed. To do this, we kept the water-to-binder ratio as 0.5 while changing the percentage of GGBFS used in the mixtures. The binder here is equal the total quantity of cement plus the total quantity of GGBFS. We also changed the percentage of recycled aggregate that replaced the normal aggregate in the mixtures. The percentages of GGBFS used were (0%, 20%, 40%, 60%, 80%), while the percentages of recycled aggregate used were (0%, 25%, 50%, 75%, 100%). The quantity of water was 175 kg and that of the superplasticizer was 2 kg per one cubic meter of concrete mixture. Figure 6 shows the general trend of increasing both GGBFS and RCA on the compressive strength in the early (7 days) and late (28 days) stages of the test (Age). As we can notice, increasing the GGBFS and RCA in the early stage led to reduction of the compressive strength values. The compressive strength of a mixture that contained 20% of GGBFS and 25% of RCA was 28.55 MPa, while the compressive strength of a mixture that contained 60% of GGBFS and 75% of RCA was 17.85 MPa. The figure also reveals that, as the GGBFS quantity is increased in the mixture, the differences in the compressive strength among mixtures with different RCA values decrease. The compressive strength of a mixture that contained 80% of GGBFS and 50% of RCA was 13.12 MPa, while the compressive strength of a mixture that contained 80% of GGBFS and 100% of RCA was 12.15 MPa. The concrete mixtures at the late stage exhibited slightly different behavior than the early stage. At higher RCA percentage values, the compressive strength of the mixture remained approximately the same across different GGBFS percentages. For example, the compressive strength of the mixtures that contained 75% of RCA and 20% of GGBFS in the mixture was 32.62 MPa, while the compressive strength of the mixtures that contained 75% of RCA and 60% of GGBFS in the mixture was 32.15 MPa.

### 4.4. Sensitivity Study

**Figure 7:**
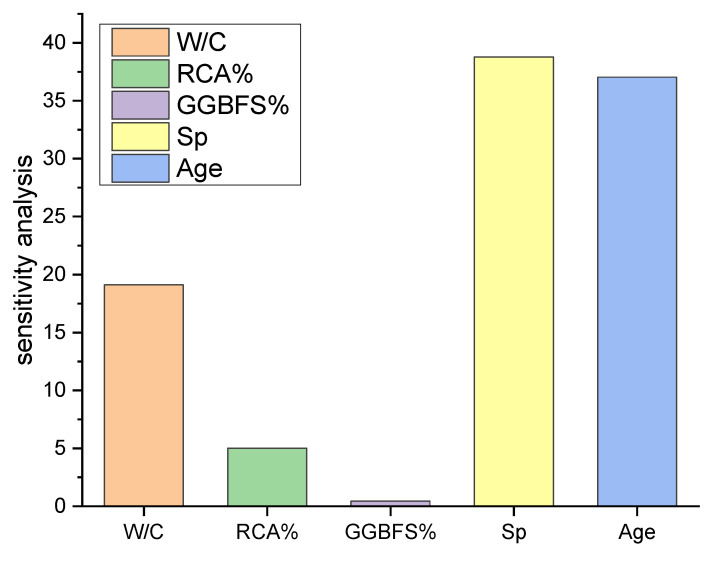
Relative importance input variables in sensitivity analysis with MPR.

The goal of this analysis was to see how input factors affect CS predictions. The influence of each input parameter on CS prediction is shown in Figure 7. This research found that Sp was the most important element, accounting for 38.77 percent of the total, followed by Age, accounting for 37.04 percent. The remaining input factors, on the other hand, had a smaller role in the prediction of CS, with W/C ratio accounting for 19.01 percent, RCA for 5.03 percent, and GGBFS for 0.45 percent. Equations (6) and (7) were used to calculate the sensitivity value (percent) of the dependent variable with respect to each independent variable [34].

(6) $$N_{i}=f_{max}\left(x_{i}\right)-f_{min}\left(x_{i}\right)$$

(7) $$S_{i}=\frac{N_{i}}{\sum_{i=1}^{n}N_{i}}\times 100$$

where $f_{max}\left(x_{i}\right)$ is the estimated output’s highest value and $f_{min}\left(x_{i}\right)$ is the anticipated output’s minimum value across the ith input domain, and all other variables are set to their mean values.

## 5. Limitation for Future Work

The dataset employed to create our prediction models was rather small, with only 125 cases. This is thus a limitation of our study; using a larger dataset can increase the precision and reliability of the MPR model. Other indications or variables may have been overlooked as a result of data collection difficulties. In supervised machine-learning-based systems, the quality and kind of data used have a substantial impact on their performance. In future research, the researchers intend to examine whether the accuracy of the proposed model can be improved by introducing a new prediction model or by increasing or decreasing the training dataset.

## 6. Conclusions

This study focused on the idea of using a white-box machine learning method, such as MPR, to build prediction equations for the compressive strength of eco-friendly concrete. A data collection of 125 recordings of concrete mix specimens was gathered to train and validate the MPR machine learning algorithm. Water-to-cement ratio (W/C), recycled aggregate percentage as a replacement material for normal aggregate in the mixture (RAC percent), GGBFS percentage as a replacement material for OPC in the binder (GGBFS percent), superplasticizer, and age were the predictors used from the literature to build the models (days). The compressive strength (CS) of eco-friendly concrete was the outcome variable. Using the cross-validation technique to evaluate our model, the MPR model showed a superior performance when compared with LR (white-box model) and SVM (black-box model) in terms of *R*^2^ and RMSE. The average prediction performances of the MPR model were 0.818 for *R*^2^ and 4.659 for RMSE, while the average prediction performances of the SVM model were 0.676 for *R*^2^ and 6.053 for RMSE across the five-fold cross-validation. LR had the poorest performance among all models. The MPR model formula obtained from the training dataset was validated with the testing dataset. The predicted values of the compressive strength of eco-friendly concrete for the testing dataset were close to the experimental values using the MPR model. Moreover, a parametric study was performed to study the effect of increasing RCA and GGBFS on the compressive strength. The study confirmed that for a higher percentage of RCA in the mixture at the late stage, the compressive strength of concrete with a high percentage of GGBFS and a low cement content is similar to that of concrete with a high content of cement and a low percentage of GGBFS.

## Figures and Tables

**Table 1:** Statistical measures on variables.

Statistics	W/C	RCA%	GGBFS%	Sp (kg)	Age (days)	CS (MPa)
Median	0.71	50	40	1.15	28	34.05
Mean	0.92	51.20	35.64	1.88	32.66	34.44
Minimum	0.4	0	0	0	7	12.4
Maximum	3.7	100	90	7.8	90	56.63
Range	3.3	100	90	7.8	83	44.23
Standard deviation	0.57	37.14	25.82	2.36	27.50	10.15

W/C = water-to-cement ratio; RCA = recycled aggregate replacement percentage; GGBFS = ground granulated blast-furnace slag replacement percentage; Sp = superplasticizers quantity in kg; Age = the time of compressive test after pouring; CS = compressive strength.

**Table 2:** Cross-validation measurement results.

Statistics	MPR		SVM		LR	
	*R* ^2^	RMSE	*R* ^2^	RMSE	*R* ^2^	RMSE
Median	0.835	4.958	0.712	6.202	0.424	7.7083
Mean	0.818	4.659	0.676	6.053	0.495	7.38138
Minimum	0.740	3.391	0.467	5.290	0.276	6.3806
Maximum	0.884	5.638	0.755	6.493	0.720	7.9816

**Table 3:** The independent variable values and the corresponding actual vs. predicted values using the MPR model.

Point	W/C	RCA%	GGBFS%	Sp (kg)	Age (days)	Actual CS (MPa)	Predicted CS (MPa)	Individual Error (MPa)
1 [28]	0.568	50	25	1.15	7	23.30	25.41	−2.11
2 [27]	0.714	25	30	3.42	28	38.80	40.03	−1.23
3 [26]	1.250	0	60	0	90	39.42	37.32	2.10
4 [26]	0.651	25	20	0	7	24.45	24.88	−0.43
5 [28]	0.852	50	50	1.15	56	25.70	34.23	−8.53
6 [28]	0.426	50	0	1.15	56	35.10	37.99	−2.89
7 [27]	1.250	25	60	3.42	7	17.20	20.68	−3.48
8 [28]	0.852	50	50	1.15	7	20.30	22.64	−2.34
9 [26]	0.833	0	40	0	90	42.78	40.51	2.27
10 [27]	1.250	100	60	3.8	7	19.40	21.56	−2.16
11 [26]	0.868	25	40	0	7	22.41	22.81	−0.39
12 [23]	1.111	0	55	0	28	42.00	34.68	7.32
13 [24]	0.464	75	15	2.28	28	41.99	39.45	2.54
14 [26]	0.689	100	20	0	28	27.54	30.01	−2.47
15 [25]	3.700	100	90	7.8	28	30.50	35.32	−4.82
16 [26]	0.833	0	40	0	28	33.26	34.80	−1.54
17 [26]	0.868	25	40	0	28	31.75	33.43	−1.68
18 [27]	0.714	100	30	3.8	7	33.30	30.66	2.64
19 [26]	0.625	0	20	0	28	34.76	34.97	−0.21
20 [23]	1.111	50	55	0	28	43.00	32.95	10.05
21 [28]	0.852	50	50	1.15	28	24.80	32.94	−8.14
22 [26]	0.833	0	40	0	7	24.19	24.56	−0.38
23 [26]	1.327	50	60	0	7	19.00	18.10	0.90
24 [24]	0.400	25	0	2.28	28	54.17	49.63	4.54
25 [25]	3.700	100	90	7.8	56	34.00	35.57	−1.57

## Data Availability

Not Applicable.
